# METS-IR/HOMA-IR and MAFLD in U.S. adults: dose–response correlation and the effect mediated by physical activity

**DOI:** 10.1186/s12902-024-01646-w

**Published:** 2024-08-01

**Authors:** Hongye Peng, Jingjing Xiang, Liang Pan, Mo Zhao, Bin Chen, Shuxia Huang, Ziang Yao, Jing Liu, Wenliang Lv

**Affiliations:** 1 Department of Infection, Guang’anmen Hospital, , China Academy of Chinese Medical Sciences, Beijing, China; 2 Graduate School, Beijing University of Chinese Medicine, 100029 Beijing, China; 3 Department of Traditional Chinese Medicine, Beijing Aerospace General Hospital, Beijing, China; 4 Phase 1 Clinical Trial Center, Deyang People’s Hospital, Deyang, Sichuan China; 5 Pharmacy Department, People’s Hospital of Mianzhu, Sichuan Mianzhu, China

**Keywords:** MAFLD, METS-IR, HOMA-IR, Physical activity, Mediating effects

## Abstract

**Objectives:**

Metabolic dysfunction-associated fatty liver disease (MAFLD), a globally prevalent disease, is closely linked to insulin resistance (IR). Physical activity (PA) is closely linked to both MAFLD and IR. We aim to explore the dose–response relationship between metabolic score for IR (METS-IR)/homeostasis model assessment of IR (HOMA-IR) and MAFLD, and investigate the relationship between PA, IR and MAFLD.

**Methods:**

Participants from the NHANES study were included in this cross-section study. Logistic regression and the receiver operating characteristic were used to assess the predictive performance of METS-IR/HOMA-IR for MAFLD. Restrictive cubic splines were performed to visualize their dose–response relationship. Decision tree analysis was used to identify high-risk populations of MAFLD. PA’s mediating effect in the association between METS-IR/HOMA-IR and MAFLD was also examined.

**Results:**

Of all 1,313 participants, 693 had MAFLD (52.78%). There were a positive association between METS-IR (OR = 1.162, 95% CI = 1.126–1.199) and HOMA-IR (OR = 1.630, 95% CI = 1.431–1.856) and MAFLD risk. The AUCs of the METS-IR and HOMA-IR were 0.831 (0.809, 0.853) and 0.767 (0.741, 0.791), respectively, with significantly different predictive performance (*P* < 0.001). Adding METS-IR/HOMA-IR to the basic model greatly improved the statistical significance for MAFLD. Five high-risk subgroups were identified for MAFLD. PA mediated about 0.81% and 0.78% (indirect effect/total effect) in the association between METS-IR/HOMA-IR and MAFLD.

**Conclusions:**

MAFLD risk might be predicted by METS-IR/HOMA-IR, among which METS-IR performed better. And PA mediated the association between them. More attention should be paid to the therapeutic effect of lifestyle changes on MAFLD.

**Highlights:**

1. Positive associations were found between METS-IR and HOMA-IR and MAFLD risk.

2. METS-IR has better predictive performance for MAFLD risk than HOMA-IR.

3.Two high-risk subgroups were identified for MAFLD by METS-IR: individuals with METS-IR ≥ 40; Hispanic black individuals with 34 ≤ METS-IR < 40 and aged ≥ 46.

4. In the significant association between METS-IR/HOMA-IR and MAFLD, about 0.81% and 0.78% (indirect effect/total effect), respectively, were mediated by physical activity.

**Graphical Abstract:**

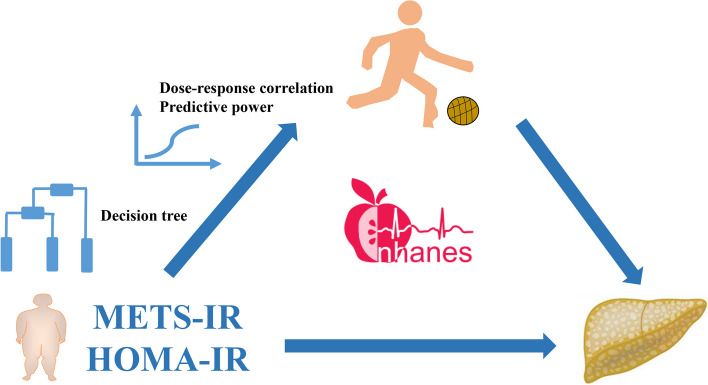

**Supplementary Information:**

The online version contains supplementary material available at 10.1186/s12902-024-01646-w.

## Introduction

As the most common chronic liver condition worldwide, non-alcoholic fatty liver disease (NAFLD) imposes substantial health and economic burdens. The prevalence rate of NAFLD is pooled as 32.4% in 2022 globally [1]. NAFLD is closely linked to various chronic metabolic diseases including type 2 diabetes mellitus (T2DM), hyperlipemia, and hyperuricemia [2, 3]. Given that NAFLD typically coexists with other liver conditions including alcoholic fatty liver and viral hepatitis, and that metabolic dysfunction contributes greatly in its pathogenesis, the 2020 International Expert Consensus recommends substituting metabolic associated fatty liver disease (MAFLD), a symptom of multisystem metabolic disorders that impact the liver, for NAFLD [4]. Previous study suggested that MAFLD, compared to NAFLD, was more closely associated with advanced liver fibrosis, cardiovascular mortality and all-cause mortality [5]. MAFLD requires more attention from researchers for early intervention to lower the risk of death. However, MAFLD is still understudied presently and needs exploration in many fields.

It is known that insulin resistance (IR), including peripheral IR and hepatic IR, is closely associated with incidence and development of MAFLD [6]. Although glucose clamp is a gold standard for sensitivity assessment to exogenous insulin [7], it is not extensively used in clinical setting as an invasive and costly approach. As a most widely used IR indicator [8], homeostasis model assessment of insulin resistance (HOMA-IR) provides quantitative evaluation of IR levels and β cell functional status, especially in the hepatic IR level. Metabolic score for insulin resistance (METS-IR), a novel indicator of insulin sensitivity proposed by Mexican researchers [9], comprises of fasting triglycerides, fasting glucose, high-density lipoprotein cholesterol (HDL-C), and body mass index (BMI). According to comparison and validation with euglycemic-hyperinsulinemic clamp, METS-IR can accurately reflect the peripheral IR level. It has been proved that METS-IR is linked to higher risks of T2DM, coronary heart disease, and hypertension [10, 11]. However, the association between MAFLD and METS-IR/HOMA-IR is unknown, so is the prediction performance of METS-IR/HOMA-IR for MAFLD.

Physical activity (PA) is a significant factor influencing both physical and mental health [12, 13]. The majority of middle-aged and older people do not get the recommended amount of PA and exercise, as physical performance declines with age [14]. Studies have suggested that insufficient PA is closely linked to MAFLD. Lack of PA or sedentariness may lead to ectopic fat accumulation, which is an independent risk factor of MAFLD [15]. Study from Korean suggests that sedentariness will cause liver damage, which increases MAFLD risk by 20% [16]. It has been concluded that MAFLD population have lower PA level compared to Non-MAFLD population [17]. And IR is strongly associated with the occurrence and development of MAFLD. IR in adipose tissue triggers lipotoxicity by increasing free fatty acids release, which accumulates toxic metabolites derived from triglycerides in ectopic tissues like the liver, contributing to MAFLD [18]. Therefore, further research is needed on the effect of PA between IR indicators and MAFLD.

Using NHANES data, we aim to investigate the dose–response correlation between MAFLD and METS-IR/HOMA-IR, as well as the predictive ability of the two indicators for MAFLD. Also, we intend to identify high risk population of MFLD with decision tree analysis. Finally, we proposed a mediating effect model to analyze the correlation between PA, IR and MAFLD for the reference of MAFLD early identification and prevention.

## Materials and methods

### Design and participants

Participants who aged 20 and above in the 2017 and 2018 cycles of NHANES were included in this cross-section study. As introduced by other researchers [19], the NHANES collected data from a nationwide non-institutionalized U.S. sample population with home visits, on-site physical checks, and lab tests. The Screening of participants was showed in Figure S1. Individuals aged under 20 (*n* = 3,955), missing MAFLD-related data (*n* = 753), taking lipid-lowering and/or hypoglycemic agents (*n* = 1,330), and with missing key data to compute METS-IR and HOMA-IR (*n* = 2,173) (Figure S1) were excluded. Eventually, 1,313 people were included out of 9,524 subjects.

### MAFLD definition

MAFLD was diagnosed with hepatic steatosis (HS) presence with ultrasonography and by satisfying a minimum of 1 criterion: being overweight/obese, having T2DM, or having a metabolic disease [20]. HS was identified by FibroScan with CAP (controlled attenuation parameters) not less than 238 dB/m because this approach demonstrated excellent accuracy in assessing hepatic steatosis level [21]. A metabolic problem was deemed to exist in normal-weight and lean participants with HS but without T2DM when any two or more metabolic anomalies were confirmed as follows: 1) waist circumference (WC) ≥ 88cm for female or 102cm for male; 2) Triglycerides (TG) level ≥ 1.70 mmol/L or medications; 3) blood pressure ≥ 130/85mmHg or medications; 4) HDL-C less than 1.3 mmol/L for female or 1.0 mmol/L for male; 5) HOMA-IR score not less than 2.5; 6) prediabetes (5.6 ≤ fasting glucose ≤ 6.9 mmol/L, or 7.8 ≤ 2h post-load glucose ≤ 11.0 mmol/L, or 5.7% ≤ hemoglobin A1c (HbA1c) ≤ 6.4%); and 7) plasma C-reactive protein > 2 mg/L.

### Measurement of METS-IR and HOMA-IR

Blood was sampled after at least 9 h of fasting. And participants went through household interviews and mobile physical examinations, including measurement of height/weight and waist circumference. All measurements were performed following applicable protocols [22]. Laboratory tests for levels of HbA1c, lipid, insulin, glucose, and C-reactive protein complied with applicable guidelines as described by CDC [23].

Indicators were calculated with the equation below [8, 9]:

METS-IR = ln (2 × FPG [mg/dL] + fasting serum triglyceride [mg/dL]) × BMI (kg/m^2^) / ln (HDL cholesterol [mg/dL]);

HOMA-IR = (FPG [mg/dL] × fasting serum insulin [μIU/mL]/405).

### Covariates

Covariates were taken from the data in household interview records such as age, gender, ethnicity, family income-poverty ratio (FIPR), and educational background (college and higher, high school, and below high school). Data of smoking, drinking per day, physical activity, and CRP were also collected. Smokers were identified as those consumed at least 100 cigarettes in the past. Nonsmokers were identified as those consumed less than 100 cigarettes or never smoked in the past. The intensity of physical activity was classified with metabolic equivalent of task (MET)-minutes/week: MET = 0 as sedentary, 0 < MET ≤ 500 as insufficient, 500 < MET ≤ 1000 as moderate, and MET > 1000 as high. All those covariates are detailed on the NHANES website, which is freely accessible to the public.

### Statistical analysis

Following NHANES guidelines for analysis, we calculated variances using clustering and stratification in the analyses, with sampling weights. Continuous variables were presented as mean ± standard deviation (SD) for normally distributed data, and as median (interquartile range, IQR) for non-normally distributed data. Categorical and dichotomous data were described using numbers and percentages. Categorical data were analyzed with the Chi-square test, while continuous data were assessed using either the t-test or Wilcoxon test, depending on the data's normality.

We computed odds ratios (OR) and 95% confidence intervals (CI) of MAFLD risk using logistic regression models, considering METS-IR/HOMA-IR as continuous variables. Model 1 examined roughly the correlation between METS-IR/HOMA-IR and MAFLD; Model 2 performed adjustment for basic covariates; Model 3 performed further adjustment for smoking/drinking, physical activity and CRP. ROC curves and AUCs were employed to evaluate the predictive power of the METS-IR/HOMA-IR for MAFLD. And their AUCs were compared using DeLong’s approach. Restrictive cubic spline analyses were performed at the 5%, 35%, 65%, and 95% of METS-IR/HOMA-IR to explore and visualize their potential association and dose–response relationship with MAFLD. Then, METS-IR/HOMA-IR were fit to a logistic regression model to confirm whether they had extra predictive ability compared to existing clinical risk factors based on C-statistics, continuous net reclassification improvement (NRI), and integrated discrimination improvement (IDI). And separate interaction analyses were completed to investigate the moderating effect of sociodemographic/behavioral covariates on the correlation between MAFLD and METS-IR/HOMA-IR. These models utilized multiplicative terms, using likelihood ratio test for interaction effect assessment. Moreover, decision tree analysis was used to identify populations at high MAFLD risk. And mediation analyses were completed to explore the effect of PA on the relationship between METS-IR/HOMA-IR and MAFLD. We computed 1000 bootstrap samples in the bootstrapped method to assess the significance of indirect effects.

Statistic analysis was performed with R 4.1. The ‘ANOVA’ function “rms” package was used to complete restricted cubic splines analysis. Decision tree analysis was completed with the 'rpart' package. Statistical significance was defined as a two-tailed *P* < 0.05.

### Ethical Statements

The protocol of NHANES was approved by the Ethics Review Board. Written informed consent was signed by each participant.

## Results

### Study population characteristics

In total, 693 participants had MAFLD (52.78%) among all the 1,313 people (Table 1). The mean age was 44 (31, 59) years [48 (35, 60) in people with MAFLD and 38.5 (28, 56) in those without]. Compared to Non-MAFLD group, MAFLD group presented higher mean age, BMI, WC, WHtR, METS-IR, HOMA-IR, FPG level, and TG level, as well as more smokers. MAFLD group had lower HDL-C level and less participants engaged in high amounts of physical activity. As for gender, height, ethnicity, educational background and drinking, MAFLD group had no statistical differences from Non-MAFLD group (Table 1).

**Table 1:** Basic characteristics of participants by Metabolic Dysfunction-Associated Fatty Liver Disease (MAFLD) in NHANES 2017–2018

Variables	Non-MAFLD (*n* = 620)	MAFLD (*n* = 693)	Total (*n* = 1313)	*P*-value
Age (years)	38.50 (28.00–56.00)	48.00 (35.00–60.00)	44.00 (31.00–59.00)	< 0.001
Gender, *n* (%)				0.140
Women	342 (55.16)	353 (50.94)	695 (52.93)	
Men	278 (44.84)	340 (49.06)	618 (47.07)	
Height(cm)	166.65 (159.30–174.13)	166.80 (159.50–174.70)	166.80 (159.30–174.40)	0.487
BMI (kg/m^2^)	24.20 (21.78- 27.52)	30.60 (27.30–35.10)	27.60 (23.90- 32.40)	< 0.001
WC (cm)	85.65 (77.98–94.45)	103.30 (95.40–114.20)	95.50 (84.60–106.90)	< 0.001
WHtR	0.52 (0.47- 0.57)	0.62 (0.57–0.68)	0.57 (0.51- 0.64)	< 0.001
Ethnicity, *n* (%)				< 0.001
Mexican American	61 (9.84)	126 (18.18)	187 (14.24)	
Non-Hispanic Black	191 (30.81)	149 (21.50)	340 (25.90)	
Non-Hispanic White	193 (31.13)	227 (32.76)	420 (31.99)	
Other	175 (28.23)	191 (27.56)	366 (27.88)	
FIPR	2.36 (1.19- 4.08)	2.06 (1.20–3.88)	2.15 (1.20–3.98)	0.445
Educational background, *n* (%)				0.188
College and higher	373 (60.16)	395 (57.00)	768 (58.49)	
High school	150 (24.19)	163 (23.52)	313 (23.84)	
Below high school	97 (15.65)	135 (19.48)	232 (17.67)	
Drinking per day	2.00 (1.00, 3.00)	2.00 (1.00, 3.00)	2.00 (1.00, 3.00)	0.108
Smoking, *n* (%)				0.701
No	384 (61.94)	421 (60.75)	805 (61.31)	
Yes	236 (38.07)	272 (39.25)	508 (38.69)	
Physical activity, *n* (%)				< 0.001
Sedentary	261 (42.10)	372 (53.68)	633 (48.21)	
Insufficient	76 (12.26)	107 (15.44)	183 (13.94)	
Moderate	74 (11.94)	54 (7.79)	128 (9.75)	
High	209 (33.71)	160 (23.09)	369 (28.10)	
FBG (mg/dL)	97.00 (93.00–103.00)	103.00 (97.00–110.00)	100.00 (95.00- 107.00)	< 0.001
TG (mg/dL)	80.50 (61.00–106.00)	109.00 (81.00–136.00)	93.00 (69.00–124.00)	< 0.001
HDL-C(mg/dL)	57.00 (48.80- 68.00)	49.00 (42.00- 58.00)	54.00 (45.00- 63.00)	< 0.001
TC(mg/dL)	180.00(159.00—207.00)	188.00 (164.00- 214.00)	184.00(162.00- 210.00)	0.002
SBP	114.66 (105.33 -126.66)	122.67(112.66- 134.66)	118.67 (109.33- 130.67)	< 0.001
DBP	70.00 (64.00—76.67)	74 .00(67.33- 80.67)	72.00 (66.00- 78.67)	< 0.001
CRP(mg/dL)	1.08 (0.58—2.46)	2.65 (1.24- 5.17)	1.70 (0.81- 3.98)	< 0.001
Hypertension, *n* (%)				< 0.001
No	528 (85.16)	458 (66.09)	986 (75.10)	
Yes	92 (14.84)	235 (33.91)	327 (24.91)	
High cholesterol, *n* (%)				0.030
No	512 (82.58)	538 (77.63)	1050 (79.97)	
Yes	108 (17.42)	155 (22.37)	263 (20.03)	
Diabetes, *n* (%)				< 0.001
No	608 (98.07)	642 (92.64)	1250 (95.20)	
Yes	12 (1.94)	51 (7.36)	63 (4.80)	
METS-IR	33.64 (29.47–39.16)	45.43 (39.79- 52.88)	39.78 (33.16–47.78)	< 0.001
HOMA-IR	1.58 (1.02–2.32)	2.90 (1.96–4.67)	2.16 (1.36- 3.58)	< 0.001

*p*-Values were calculated from chi-square tests (categorical variables) or rank-sum tests (continuous variables without normal distribution), or anova (continuous variables with normal distribution)

*MAFLD* metabolic dysfunction-associated fatty liver disease, *BMI* body mass index, *WC* waist circumference, *WHtR* waist-to-height ratio, *FIPR* family income-poverty ratio, *FBG* fasting blood glucose, *TG* Triglycerides, *METS-IR* metabolic score for insulin resistance, *HOMA-IR* homeostatic model assessment for insulin resistance, *TC* total cholesterol, *SBP* systolic blood pressure, *DBP* diastolic blood pressure, *CRP* C-reactive protein

### The level of METS-IR/HOMA-IR and the risk of MAFLD

Table S1 presented ORs and 95% CIs for the correlation between increment of METS-IR/HOMA-IR level and MAFLD risk after adjustment of multiple covariates. When all covariates were adjusted, both METS-IR (OR = 1.161, 95% CI = 1.125–1.197) and HOMA-IR (OR = 1.603, 95% CI = 1.431–1.818) were positively correlated with MAFLD risk.

### Comparing METS-IR and HOMA-IR in predicting MAFLD

**Fig. 1:**
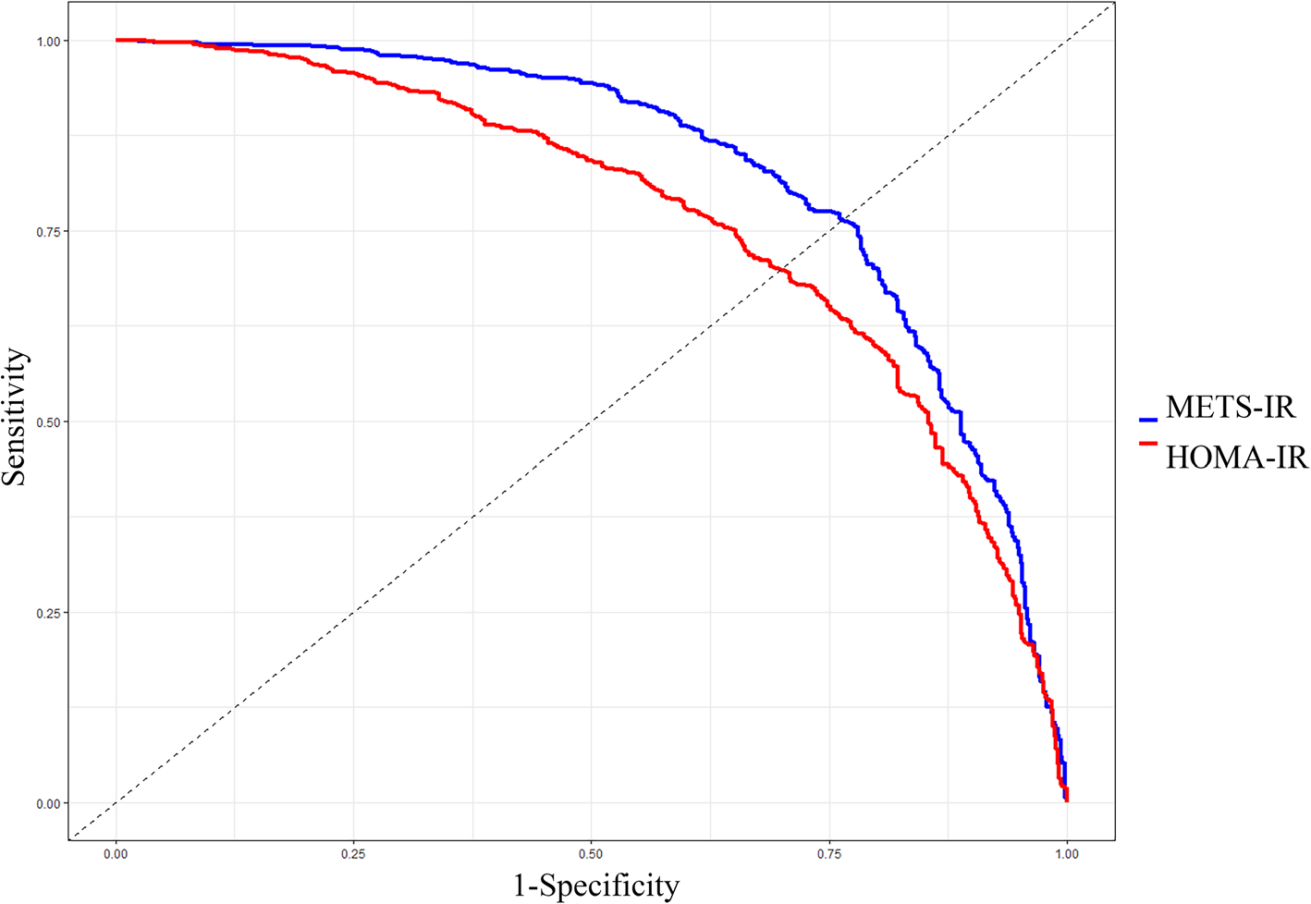
Receiver operating characteristic curves of METS-IR and HOMA-IR for identifying MAFLD. This figure demonstrates that METS-IR has better diagnostic ability for MAFLD compared to HOMA-IR

The AUCs of METS-IR and HOMA-IR were 0.831 (0.809, 0.853) and 0.767 (0.741, 0.791) respectively, indicating significantly different predictive power (*P* < 0.001) (Fig. 1). The cut-off points with METS-IR and HOMA-IR were 39.769 and 2.243, with corresponding AUCs of 0.767 and 0.703, respectively.

### Dose–response correlation between METS-IR/HOMA-IR and MAFLD

**Fig. 2:**
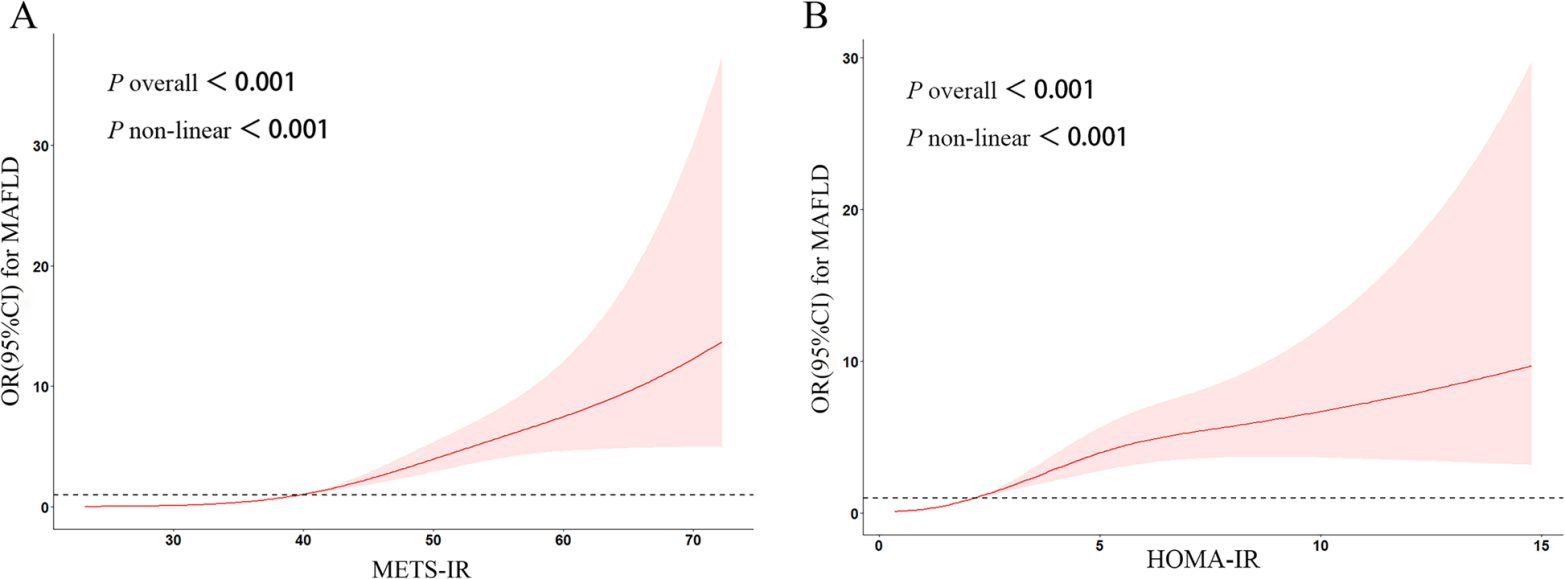
Dose–response relationships between METS-IR (**A**) /HOMA-IR (**B**) levels and MAFLD risk with restricted cubic splines. Gender, age, race, PIR, education level, smoking status, alcohol consumption, and physical activity were adjusted. The plot shows that was METS-IR and HOMA-IR was positively associated with MAFLD

Figure 2A presented the dose–response correlation between METS-IR and MAFLD. METS-IR was found to be positively correlated to MAFLD (*P* for overall < 0.001, and *P* for non-linear < 0.001). METS-IR could be protective against MAFLD at lower level (OR < 1), but such protective effect declined at a higher level. Elevated METS-IR level would increase the MAFLD risk when above a particular threshold (OR > 1). Similar findings were observed regarding the link between HOMA-IR and MAFLD (Fig. 2B).

### Increased predictive power of METS-IR and HOMA-IR

Table S2 showed that adding METS-IR to the basic model enhanced greatly the C-statistic for MAFLD (from 0.691 to 0.853, *P* < 0.001) and increased significantly the continuous NRI and IDI (by 1.024 and 0.279, respectively, *P* < 0.001). Adding HOMA-IR to the basic model enhanced the ability of MAFLD prediction, according to C-statistic increase (from 0.691 to 0.791, *P* < 0.001) as well as continuous NRI (0.758, *P* < 0.001) and IDI (0.139, *P* < 0.001) increase.

### Stratified analysis

To investigate potential subgroup differences for the association between MAFLD risk and METS-IR/HOMA-IR, we stratified participants into different subgroups (Figure S2). Results showed no significant interactions between METS-IR/HOMA-IR and age, gender, FIPR, ethnicity, educational background, smoking, drinking per day, and physical activity (*P* > 0.05).

### Decision tree analysis

**Fig. 3:**
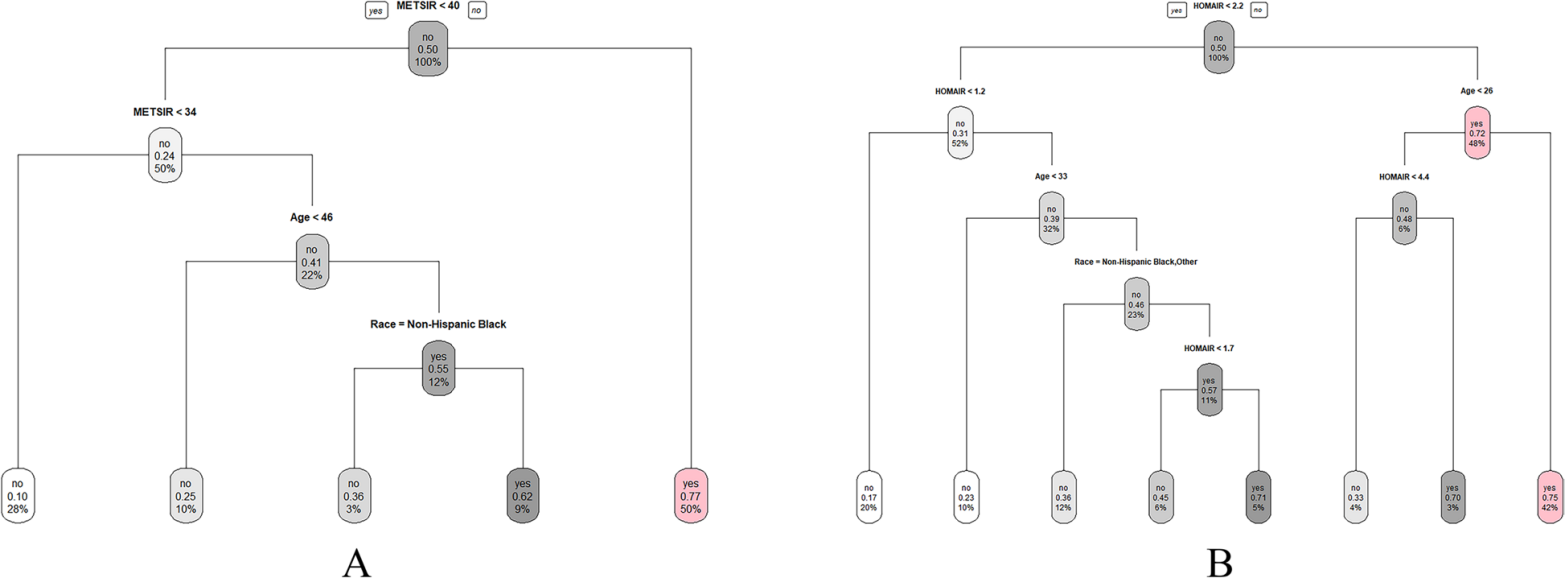
Decision Tree Model Identify Risk Groups with MAFLD. **A**: METS-IR; **B**: HOMA-IR. The plot shows MAFLD was influenced by METS-IR and HOMA-IR

Decision tree analysis results for MAFLD were shown in Fig. 3. MAFLD was influenced by METS-IR, age, and ethnicity, among which METS-IR served as the root in the model (Fig. 3A). Two high-risk subgroups were identified for MAFLD: individuals with METS-IR ≥ 40; Hispanic black individuals with 34 ≤ METS-IR < 40 and aged ≥ 46.

And MAFLD was influenced by HOMA-IR, age, and ethnicity, among which METS-IR served as the root in the model. Three high-risk subgroups were identified for MAFLD: Mexican American or Non-Hispanic White individuals with 1.7 ≤ HOMA-IR < 2.2 and aged ≥ 33; individuals with HOMA-IR ≥ 4.4 and aged < 26; and individuals with HOMA-IR ≥ 2.2 and age ≥ 26 (Fig. 3B).

### Mediation analyses

Mediation analysis results suggested that physical activity mediated the association between MAFLD and METS-IR/HOMA-IR, with indirect effect estimates and 95%CI of 3.38E-06 (1.81E-07, 0.000) and 7.50E-04 (6.21E-05, 0.000), respectively. In the significant correlation between METS-IR/HOMA-IR and MAFLD, about 0.73% and 0.67% (indirect/total effect) were mediated by physical activity, respectively (Table 2).

**Table 2:** The mediation analyses between METS-IR/HOMA-IR and MAFLD through physical activity

		Estimate	95%CI Lower	95%CI Upper	*P* -value
METS-IR-physical activity-MAFLD	ACME (average)	0.000003	0.000000	0.000000	0.040
	ADE (average)	0.000419	0.000217	0.000000	< 0.001
	Prop. Mediated	0.007350	0.000425	0.020000	0.040
HOMA-IR-physical activity-MAFLD					
	ACME (average)	0.000750	0.000062	0.000000	0.028
	ADE (average)	0.099000	0.087500	0.110000	< 0.001
	Prop. Mediated	0.006750	0.000647	0.020000	0.028

*ACME* average causal mediation effect, *ADE* average direct effect

## Discussion

As a manifestation of multisystem metabolic disorders affecting the liver, MAFLD has affected the health of one third of global population [1]. Based on a large cross-sectional study, we analyzed the characteristics of patients with MAFLD. For the exploration of IR’s role in MAFLD pathogenesis, we further analyzed the dose–response correlation between two IR indicators (METS-IR/HOMA-IR) and MAFLD risk. Results showed that METS-IR/HOMA-IR were closely linked to MAFLD risk and presented excellent predictive power for MAFLD. And we found that physical activity mediated the correlation between METS-IR/HOMA-IR and MAFLD risk.

Firstly, a positive relationship was found between METS-IR/HOMA-IR and MAFLD risk. Both obesity and IR are important factors of MAFLD. The prevalence rate of NAFLD in T2DM patients could be up to 55.5% [24]. Insulin may inhibit the lipolysis in adipocytes [25], whereas IR may increase the free fatty acids (FFA) release from adipose tissue. Excessive fatty acid release may cause lipotoxicity, resulting in buildup of triglyceride-derived harmful metabolites in ectopic tissue such as liver [18]. And circulating FFA may trigger the proinflammatory nuclear factor-kappaB pathway [26], which contributes to de novo lipogenesis (DNL) and synthesis of cholesterol, causing hepatic buildup of triglycerides and cholesterol [27] and the occurrence of MAFLD.

Furthermore, we compared the differences between METS-IR and HOMA-IR in the predictive ability for MAFLD. Interestingly, METS-IR presented better predictive ability for MAFLD compared to conventional HOMA-IR. Insulin resistance has two types, namely peripheral IR in skeletal muscle and hepatic IR in adipose tissue. HOMA-IR reflects only the hepatic insulin sensitivity [28], while METS-IR integrates BMI, FPG, TG level, HDL-C level and may better reflect the systemic functions in metabolism. Previous study suggests that MAFLD is closely linked to overweight/obesity, hypertriglyceridemia and decreased HDL-C levels [29]. Moreover, Qureshi et al. [30] found that systemic IR level was significant higher in fatty liver group and NASH (NAFLD) group, while only hepatic IR was higher in NASH group, compared to normal group. Our findings found that METS-IR had better predictive power than HOMA-IR, suggesting that peripheral IR played an indispensable role in incident MAFLD or, might due to, that patients with fatty liver outnumbered those with NASH in our study. It was noteworthy that the optimal HOMA-IR cut-off in MAFLD prediction was 2.2, which was lower than 2.5, a recognized clinical cut-off [31]. This suggests that the HOMA-IR cut-off may need down regulation to help early prevention of metabolic diseases such as MAFLD.

Moreover, dose–response analysis was performed to present objectively and comprehensively the correlation between METS-IR/HOMA-IR and MAFLD risk. Results showed that MAFLD risk increased with elevated METS-IR/HOMA-IR levels, which was similar to previous study. Triglyceride-glucose (TyG) is also an important index of IR level [32] and positively & linearly related to NAFLD risk, according to Ling et al. [33]. A Korean prospective cohort study with 8,360 samples [34] suggested that METS-IR had positive dose–response relationship with incident NAFLD, which was similar to our findings; and that HOMA-IR had a J-shaped relationship with NAFLD incidence, which was different from the positive nonlinear correlation that we found. Such difference might come from participant diversity, different diagnostic criteria for NAFLD/MAFLD, and selection of covariates. Thus, we are aware that MAFLD risk increases with elevated METS-IR/HOMA-IR levels when the latter exceeds a specific threshold, with dose-related cumulative effect. Based on those findings, we further included METS-IR/HOMA-IR in the basic model (Model 3) and found that model’s predictive power was significantly enhanced, which suggested that METS-IR and HOMA-IR were important indicators in prediction of MAFLD risk.

Stratified analysis was performed to explore possible differences in the correlation between METS-IR/HOMA-IR and MAFLD risk in different subgroups. There was no interaction between METS-IR/HMOA-IR and participants’ socioeconomic or lifestyle variables. But it was notable that the interaction between METS-IR and gender presented a *P* = 0.051 (close to *P* = 0.05), suggesting that men might be more likely influenced by higher IR level compared to women. More and more evidence suggests that gender contributes greatly in the occurrence of metabolic abnormalities, with women experiencing greater protection than men, possibly due to female sex hormones (estrogens) [35]. According to recent research, 17β-oestradiol shields pro-opiomelanocortin (POMC) neurons from developing IR [36]. Estrogens may ameliorate IR and metabolic disorders by reducing food intake, increasing energy expenditure and improving adipose tissue distribution [37].

To facilitate quick and easy identification of population at high MAFLD risk in clinical practice, we found 5 high risk subgroups using decision tree analysis: (1) individuals with METS-IR ≥ 40; (2) Hispanic black individuals with 34 ≤ METS-IR < 40 and aged ≥ 46; (3) Mexican American or Non-Hispanic White individuals with 1.7 ≤ HOMA-IR < 2.2 and aged ≥ 33; (4) individuals with HOMA-IR ≥ 4.4 and aged < 26; and (5) individuals with HOMA-IR ≥ 2.2 and aged ≥ 26. Our findings suggested that METS-IR/HOMA-IR level, age and ethnicity might be important factors for the risk of MAFLD, and METS-IR/HOMA-IR is even more important among them.

It is notable that physical activity mediates the association between IR indicators and MAFLD, which means that, PA has impact on MAFLD by affecting IR. Growing high-quality evidence suggests that proper PA can significantly improve the local and even systemic effects of many different diseases [38–40]. Physical activity and exercise may reduce inflammation and postpone the progression of obesity-related complications [41]. And IR can be significantly improved by reducing inflammation and losing weight [42]. Compared to population with insufficient PA, those with proper PA have lower IR level [43]. Researchers suggested that proper PA can significantly lower MAFLD risk as well as liver fibrosis and cirrhosis risks [44, 45]. Moreover, higher energy expenditure and higher exercise intensity, such as high-intensity interval training, offer greater benefits for improving overall insulin sensitivity. Both aerobic exercises (e.g., walking, cycling) and resistance training can improve glycemic control and insulin sensitivity, with combined exercise programs potentially providing the optimal effect [46, 47]. Our study also proves that enhanced PA can lower the risk of MAFLD by improving IR. Therefore, maintaining proper PA is important for healthy lifestyle, which is worthy of our attention in the future.

This study has several advantages. First, our findings come from a large-scale study across the United States with representative sample. Second, peripheral IR contributes greatly in the incidence and development of MAFLD, according to the comparison of predictive power between METS-IR and HOMA-IR. Third, populations at high risk of MAFLD are identified with decision tress analysis for the reference of MAFLD screening in clinical practice. And last, PA mediates the association between IR indicators and MAFLD risk, which confirms the important role of a healthy lifestyle in improving MAFLD.

Also there are some limitations. Firstly, cause-and-effect correlation between METS-IR/HOMA-IR and MAFLD risk can not be confirmed in our cross-sectional study. Secondly, transient elastography was used to diagnose HS instead of a histological gold standard. Third, despite of adjustment for many covariates, there were other possible confounders, like diet. And last, this study only focuses on populations in the United States, so more clinical research is needed to confirm the applicability of our findings to other ethnicities.

## Conclusion

In conclusion, we suggest that the risk of MAFLD can be possibly predicted with METS-IR/HOMA-IR, among which METS-IR has better predictive power. And PA mediates the association between IR indicators and MAFLD risk, so more focus should be placed on the therapeutic impact of lifestyle changes on MAFLD. Although MAFLD exerts increasing burden on global healthcare, present therapy is mostly limited to lifestyle improvement and weight loss instead of effective drugs. Early identification of populations at high risk of MAFLD may relief global burden and has public health significance in disease prevention. Our findings confirm that enhancing insulin sensitivity, especially peripheral insulin sensitivity, and proper physical activity, are important measures for MAFLD prevention.

### Supplementary Information

Additional file 1: Table S1. Multi-variate adjusted odds ratio (95% CIs) for the relationship between the risk of MAFLD and increment of METS-IR/HOMA-IR level among participants.

## Data Availability

Publicly accessible datasets from the Centers for Disease Control and Prevention were utilized in this study. The specific information can be found in the National Health and Nutrition Examination Survey (NHANES) report released by the U.S. Department of Health and Human Services in 2017. Available from https:// wwwn.cdc.gov/nchs/data/nhanes/2017–2018/manuals/2017_MEC_ Laboratory_Procedures_Manual.pdf accessed 31 March 2020.

## Abbreviations

BMI: Body mass index
CAP: Controlled attenuation parameters
CI: Confidence intervals
FFA: Free fatty acids
FIPR: Family income-poverty ratio
HbA1c: Hemoglobin A1c
HDL-C: High-density lipoprotein cholesterol
HOMA-IR: Homeostasis model assessment of insulin resistance
HS: Hepatic steatosis
IDI: Integrated discrimination improvement (IDI)
IR: Insulin resistance
MAFLD: Metabolic dysfunction-associated fatty liver disease
MET: Metabolic equivalent of task
METS-IR: Metabolic score for insulin resistance
NAFLD: Non-alcoholic fatty liver disease
NRI: Net reclassification improvement
OR: Odds ratios
PA: Physical activity
T2DM: Type 2 diabetes mellitus
TG: Triglycerides
TyG: Triglyceride-glucose
WC: Waist circumference
