# Supplementary material for: METS-IR/HOMA-IR and MAFLD in U.S. adults: dose–response correlation and the effect mediated by physical activity
Source: BMC Endocr Disord. 2024 Aug 1;24:132. doi: 10.1186/s12902-024-01646-w (PMC11293050; doi:10.1186/s12902-024-01646-w)
Supplement: Supplementary file 1 — Additional file 1: Table S1. Multi-variate adjusted odds ratio (95% CIs) for the relationship between the risk of MAFLD and increment of METS-IR/HOMA-IR level among participants. [file 12902_2024_1646_MOESM1_ESM.docx]

**Supplementary Material**

**Table S1 Multi-variate adjusted odds ratio (95% CIs) for the relationship between the risk of MAFLD and increment of METS-IR/HOMA-IR level among participants**

| Variables | OR (95% CI) | *P*-vaule |
| --- | --- | --- |
| METS-IR (per 1 increment) |  |  |
| Model 1 | 1.153(1.121,1.187) | ＜0.001 |
| Model 2 | 1.153(1.126,1.201) | ＜0.001 |
| Model 3 | 1.161(1.125,1.197) | 0.011 |
| HOMA-IR (per 1 increment) |  |  |
| Model 1 | 1.595(1.378,1.846) | ＜0.001 |
| Model 2 | 1.595(1.426,1.881) | ＜0.001 |
| Model 3 | 1.603(1.413,1.818) | 0.018 |

Note:All estimates accounted for complex survey designs. Model 1 included only independent variables; model 2 was additionally adjusted for gender, age, ethnicity, FIPR and education level; and model 3 was further adjusted for smoking status, alcohol consumption, physical activity and CRP.

**Table S2 Incremental predictive value of METS-IR and HOMA-IR**

|  |  |  |  |  |  |  |
| --- | --- | --- | --- | --- | --- | --- |
|  | C statistic  **Estimate (95% CI)** | ***P*-value** | **NRI (continuous)**  **Estimate (95% CI)** | ***P*-value** | **IDI**  **Estimate (95% CI)** | ***P*-value** |
| METS-IR |  |  |  |  |  |  |
| Basic model | 0.691(0.691,0.791) |  | **ref** |  | **ref** |  |
| Basic model + METS-IR | 0.853(0.833,0.874) | <0.001 | 1.024(0.932,1.117) | <0.001 | 0.279(0.256,0.303) | <0.001 |
| HOMA-IR |  |  |  |  |  |  |
| Basic model | 0.691(0.691,0.791) |  | **ref** |  | **ref** |  |
| Basic model + HOMA-IR | 0.791(0.767,0.815) | <0.001 | 0.758(0.661,0.855) | <0.001 | 0.139(0.121,0.157) | <0.001 |

Note: We incorporate METS-IR/HOMA-IR into a basic logistic regression model to determine whether they have more predictive value. The basic model included gender, age, ethnicity, FIPR and education level; smoking status, alcohol consumption, physical activity and CRP.

Abbreviations：NRI, net reclassification improvement; IDI, integrated discrimination improvement.


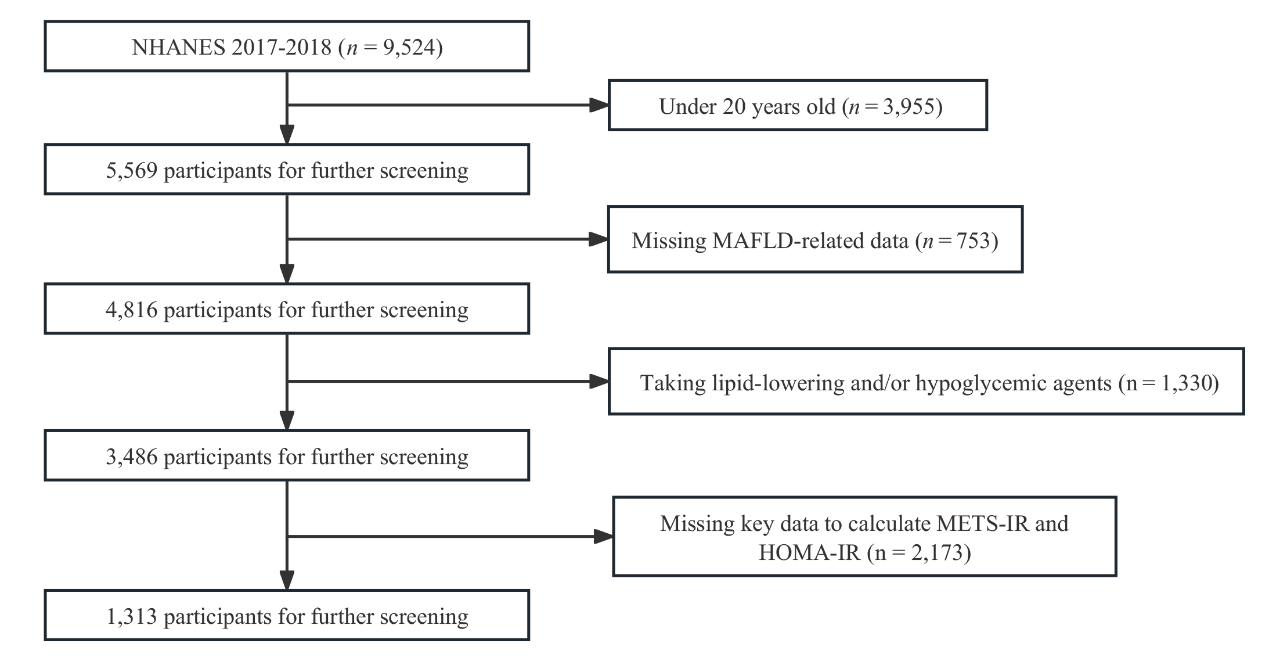


**Figure S1 Flow chart for the selection of participants in the cross study.**


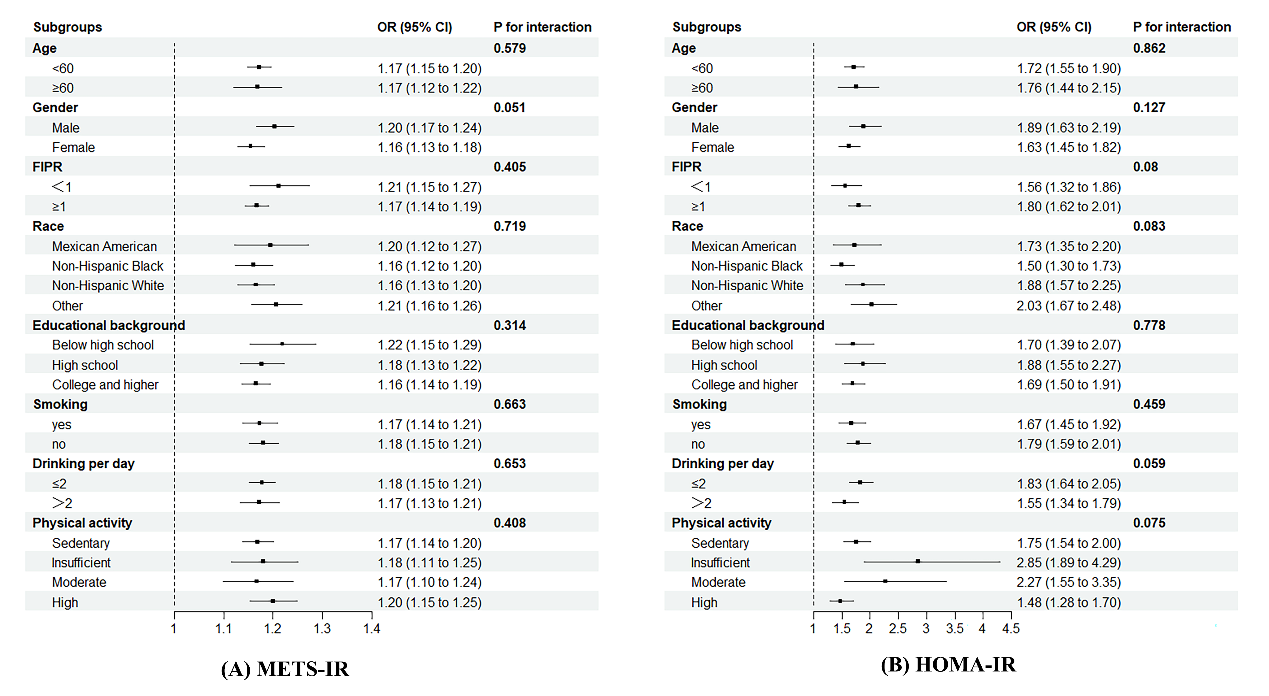


**Figure S2 Forest plot of stratified analysis of the association of METS-IR (A) and HOMA-IR (B) index with the risk of MAFLD.**
